# How to improve the COVID-19 health education strategy in impoverished regions: a pilot study

**DOI:** 10.1186/s40249-022-00963-3

**Published:** 2022-03-29

**Authors:** Huimin Wang, Rujun Liao, Xin Chen, Jie Yu, Tianyu Zhu, Qiang Liao, Tao Zhang

**Affiliations:** 1 Department of Epidemiology and Health Statistics, West China School of Public Health and West China Fourth Hospital, Sichuan University, Chengdu, Sichuan China; 2 Sichuan Center for Disease Control and Prevention, Chengdu, Sichuan China; 3 Liangshan Prefecture Center for Disease Control and Prevention, Xichang, Sichuan China

**Keywords:** COVID-19, Health education strategy, Impoverished region, Health literacy, Structural equation model, Mediating effect, Moderating effect

## Abstract

**Background:**

It is of great challenge to raise the public coronavirus disease 2019 (COVID-19) related health literacy (CRHL) in impoverished regions due to the limits of poor infrastructure, large proportion of vulnerable groups, etc. However, those limits cannot be solved in the short term. Therefore, this study chose Liangshan Yi Autonomous Prefecture, one of the poorest areas in China, as a pilot, to reveal the quantitative relationships among different dimensions under the COVID-19 health education framework, clarify the key points for health promotion, and provide specific suggestions for COVID-19 health education strategy in impoverished regions.

**Methods:**

A cross-sectional questionnaire survey was conducted in five regions of Liangshan Yi Autonomous Prefecture in 2020. There were 2,100 individuals sampled by multi-stage method. This survey mainly measured the four dimensions: CRHL, COVID-19 related tense psychological reactions (CRTPR), COVID-19 related information report acquisition (CRIRA), and general health literacy (GHL). The multivariate logistic regression was used to explore the influence of demographic characteristics on each dimension. Furthermore, to quantify the relationships among different dimensions, this study employed the structural equation model (SEM), and analyzed the mediating effects of CRHL and CRIRA as well as the moderating effects of regional characteristic variables.

**Results:**

The CRHL played an important role in promoting COVID-19 health education, reaching 52.5% in Liangshan Yi Autonomous Prefecture. The GHL (*β* = 0.336) and age (*β* = 0.136) had statistically positive impacts on CRHL. The CRHL affected CRTPR negatively (*β* = − 0.198) and CRIRA positively (*β* = 0.052). The CRHL played significant mediating roles among the four dimensions (*P* < 0.05). Effectiveness of government prevention and control as well as the ethnicity moderated not only the relationships between CRHL and other dimensions, but also the mediating effect of CRHL (*P* < 0.05). People with lower income and education levels had lower GHL (*β* = 0.286, 1.292). The youth were more likely to show CRTPR (*β* = − 0.080).

**Conclusions:**

By proposing and verifying the theoretical framework, this study put forward specific suggestions on how to improve COVID-19 health education strategies in impoverished regions via *implementation methods*, *key groups* and *effect evaluation*, which also provided references about future public health emergencies for other impoverished regions of the world.

**Graphical Abstract:**

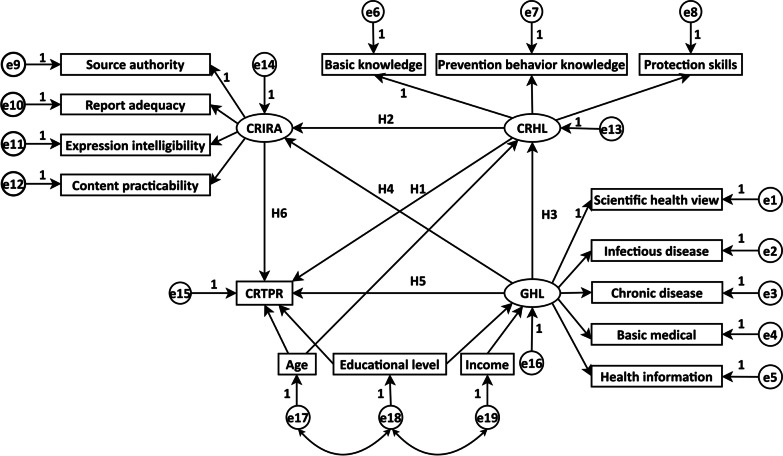

**Supplementary Information:**

The online version contains supplementary material available at 10.1186/s40249-022-00963-3.

## Background

The coronavirus disease 2019 (COVID-19) pandemic posed a serious threat to human health, especially in impoverished regions, which signaled a strong warning. The impoverished regions have backward economic conditions and weak health systems, and face the double burden of infectious and noninfectious diseases. Furthermore, it was reported that the public’s awareness of the epidemic and health literacy were often low in impoverished regions around the world, such as some areas in Kenya and Sudan, accompanied by a large number of widespread misunderstandings and misinformation about the COVID-19 [1, 2]. Those misconceptions are conceivable threats to COVID-19 management strategies. Therefore, public health literacy is crucial for fighting against the epidemic. A good level of COVID-19 related health literacy (CRHL) is essentially necessary for full compliance of the public with prevention and control measures, which can help people identify the fake news, trigger the public’s early and immediate response to the epidemic, and promote people to take proactive preventive measures in the face of the future epidemic. As a result, it is necessary to pay special attention to how to improve COVID-19 health education in impoverished regions.

Although there have been some previous researches on promoting CRHL, they were mostly focused on developed countries [3–5], or well-educated populations in developing countries, such as college students and medical workers [6–8]. Correspondingly, the majority of measures proposed to enhance CRHL were based on the Internet, or to improve information transparency and optimize official communication [9, 10]. However, there were few studies on CRHL in impoverished regions. In fact, there are a large proportion of vulnerable groups in impoverished regions, such as the low-income and low-educated classes, who are the most vulnerable to the impact of epidemic. However, due to the divergence of demographic characteristics, science and technology, economy, and culture, those existing research strategies aimed at developed countries or well-educated populations could not be fully applied to impoverished regions.

Therefore, this study chose Liangshan Yi Autonomous Prefecture as the pilot, which was a typical representative of impoverished areas in China. Before the year of 2021, there were 11 of the 17 counties which were deeply impoverished. It is the largest Yi-inhabited area. Meanwhile, with the characteristics of ancient and closed, it was also one of the areas where slavery was finally eliminated in China. Coupled with factors such as inconvenient transportation, inclement weather, backward infrastructure, and weak public health conditions, it has always been the main battlefield for the prevention and control of infectious diseases, thus facing severe challenges of COVID-19 epidemic [11]. After the outbreak of COVID-19, referring to the 2020 national resident health literacy monitoring questionnaire issued by the National Health Commission of China, Liangshan Yi Autonomous Prefecture put forward the COVID-19 health education framework consisted of four dimensions: CRHL, COVID-19 related tense psychological reactions (CRTPR), COVID-19 related information report acquisition (CRIRA), and general health literacy (GHL). To this end, this study aimed to reveal the quantitative relationships among different dimensions, clarify the key points for health promotion, and provide specific suggestions for COVID-19 health education strategy in impoverished regions to help better respond to the epidemic.

## Methods

### Data collection and preprocessing

This study adopted the 2020 national resident health literacy monitoring questionnaire issued by the National Health Commission of China to the Centers for Disease Control and Prevention at all levels. It consisted of two parts, and one was the annually-updated general health literacy of residents, which was utilized by this study to measure the GHL. The other part was the special questionnaire for COVID-19, which was newly added in 2020 due to the outbreak of COVID-19. This study adopted this part to measure the CRHL, CRIRA, and CRTPR. According to the correct answers of the questionnaire, the respondent whose questionnaire score reached 80.0% or more of the total score was judged to be health literate, and the regional level of health literacy referred to the proportion of people with health literacy in the total population [12]. The definitions of the four dimensions (CRHL, CRTPR, CRIRA, and GHL), as well as other details, were listed in Table 1.

**Table 1:** Dimension structure, definition and measurement description of the questionnaire

Dimension	Definition	Manifest variable	Measurement description
CRHL	The health literacy related to COVID-19	Basic knowledge of COVID-19	Sum of 13 yes/no items^a^
		Prevention behavior knowledge of COVID-19	Sum of 12 yes/no items
		Protection skills of COVID-19	Sum of 16 yes/no items
GHL	The ability of individuals to obtain, understand and process general health information and services, and use them to make decisions conducive to improving and maintaining their own health	Scientific health view	Sum of 21 yes/no items
		Infectious disease prevention and control literacy	Sum of 11 yes/no items
		Chronic disease prevention and control literacy	Sum of 27 yes/no items
		Basic medical literacy	Sum of 23 yes/no items
		Health information literacy	Sum of 15 yes/no items
CRIRA	The evaluation of respondents on the access to COVID-19 related information report	Information source authority	5-point Likert-type scale^b^
		Information reporting adequacy	5-point Likert-type scale
		Information expression intelligibility	5-point Likert-type scale
		Information content practicability	5-point Likert-type scale
CRTPR	The respondents’ anxiety and tense psychological reactions related to COVID-19	CRTPR	Sum of two 5-point Likert-type scales and 7 yes/no items^c^

^a^Scored as 1 = yes, 0 = no. ^b^Scored as 1 = disagree, 5 = strongly agree. ^c^Scored as 0 = not at all, 1 = strongly nervous, 0 = no, 1 = yes. *CRHL* COVID-19 related health literacy, *GHL* general health literacy, *CRIRA* COVID-19 related information report acquisition, *CRTPR* COVID-19 related tense psychological reactions

This study carried out a cross-sectional survey from August 14, 2020 to December 11, 2020 in Liangshan Yi Autonomous Prefecture, which was part of the national resident health literacy continuous monitoring survey conducted annually since 2012 [12]. The multi-stage sampling framework was used to select individual respondents. Firstly, according to the economic and social development status and ethnic distribution, five representative regions of Liangshan Yi Autonomous Prefecture were selected, namely Huili County, Muli County, Xichang City, Yuexi County, and Zhaojue County. Then the probability proportionate to size sampling (PPS) method was adopted to draw four towns or streets from each region, and three villages or communities from each town or street. There were 35 households randomly chosen from each village or community. Besides, one permanent resident aged 15 to 69 was selected from each household by the Kish table [13]. Finally, there were 2100 individuals sampled by this study.

Since some respondents had too low literacy to complete the questionnaire by themselves, this study conducted a household questionnaire survey of face-to-face interviews to ensure the survey quality. In order to effectively control the survey quality and facilitate the follow-up study, the name of respondent was mandatory. Considering privacy protection, both respondents and investigators signed informed consents, which emphasized the confidentiality of the respondents’ information and explained the purpose, importance, and main content of the investigation to the respondents. The ethics committee at Liangshan Prefecture Center for Disease Control and Prevention approved this study (LS2020005).

Quality control was carried out at the pre-investigation, investigation, and data processing stages. At the pre-investigation stage, all members in the study team received strict professional training followed by a preliminary investigation. When it came to the investigation stage, the research associates checked the questionnaires every day and called for amendments if any violation of the study protocol was detected. Finally, during the data processing stage, a double-recording comparison was used for data input, and complete case analysis was adopted to deal with logical errors, outliers and missing data problems [14].

### Analyzing the influence of demographic characteristics on each dimension

There may be differences in CRHL, CRTPR, CRIRA, and GHL among people with different demographic characteristics. Some studies have reported that education, race/ethnicity, age, income, occupation, gender, etc. were associated with health literacy or psychological distress related to COVID-19 [15–17]. Therefore, it was necessary to explore whether different characteristics of people in impoverished regions have statistically significant impacts on the levels of the above four dimensions, so as to make policies, strategies, and measures more targeted and accurate.

To explore whether the levels of CRHL, CRTPR, CRIRA, and GHL were different among people with different demographic characteristics, we conducted the multivariate logistic regression analysis. Demographic characteristics were taken as independent variables. With GHL, CRHL, CRIRA, and CRTPR as study outcomes respectively, the medians of their measures were taken as the cut-off points [18]. According to the hypotheses below, the GHL was covariate of the model with CRHL as the study outcome. Similarly, the GHL and CRHL were covariates of the CRIRA model. The GHL, CRHL, and CRIRA were covariates of the CRTPR model. The stepwise regression method was adopted to explore the demographics that had statistical significance on each dimension.

### Exploring relationships among different dimensions

**Fig. 1:**
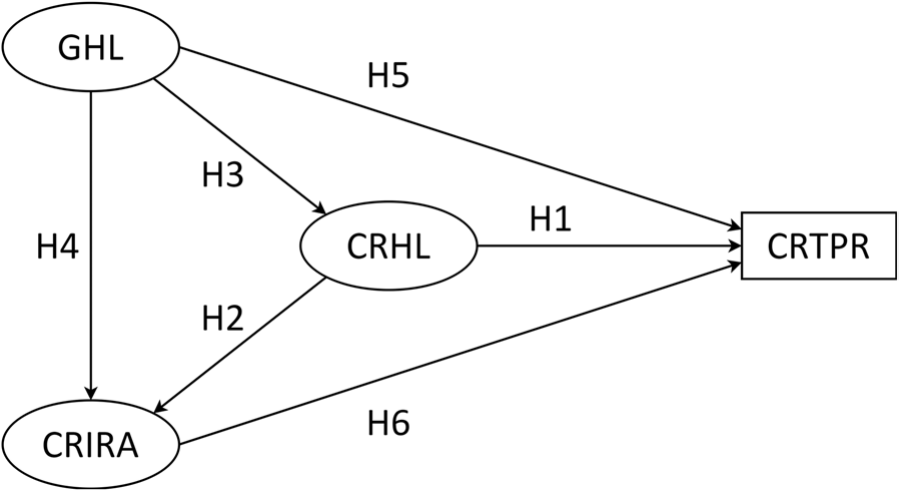
Theoretical framework of the direct relationships among the four dimensions (CRHL, CRTPR, CRIRA, and GHL). The ellipses represented the latent variables, and the rectangle represented the manifest variable. *CRHL* COVID-19 related health literacy, *GHL* general health literacy, *CRIRA* COVID-19 related information report acquisition, *CRTPR* COVID-19 related tense psychological reactions

In order to improve the COVID-19 health education strategy, one important step was to reveal the quantitative relationships among different dimensions under the COVID-19 health education framework. Regarding the literature reviews and related hypotheses mentioned below, this study proposed the theoretical framework of the relationships among the four dimensions (CRHL, CRTPR, CRIRA, and GHL), as shown in Fig. 1. Due to the aim of this study, these relationships could be classified as three parts, i.e., direct effects involving CRHL, direct effects not involving CRHL, and indirect effects among dimensions.

#### Hypotheses on direct effects involving CRHL

Figure 1 showed that increase of CRHL may reduce CRTPR, that was:

**H1**: *CRHL negatively affects CRTPR*.

People with higher CRHL were less likely to experience excessive tension because they had a better understanding of COVID-19 related health information. Researchers revealed that the knowledge or confidence of the COVID-19 epidemic showed an inverse correlation with psychological distress [19, 20]. To this end, this study put forward the first hypothesis above.

In addition, Fig. 1 also indicated that there may be two other dimensions directly associated with CRHL. Firstly, the high uncertainty of an unknown disease may arouse people to try reducing uncertainty by obtaining related information to make sound decisions. People with high CRHL may actively and intensively search for disease-related information. There were studies that indicated a clear association between misinformation belief and poorer COVID-19 knowledge [21, 22]. Therefore, we proposed the following hypothesis:

**H2**: *CRHL positively affects CRIRA*.

Another associated factor with CRHL was GHL. Generally, individuals with higher GHL level had more knowledge about infectious diseases. Research has shown that confusion about COVID-19 was significantly higher among those who had lower health literacy [4]. Hence, it came into being the hypothesis as followed:

**H3**: *GHL positively affects CRHL*.

#### Hypotheses on direct effects not involving CRHL

Furthermore, it has been highlighted that health education and improvement of health literacy were critical prevention and health promotion measures for mitigating the adverse effects of the so called *infodemic* [23, 24]. On these grounds, we proposed that:

**H4**: *GHL positively affects CRIRA*.

In addition, groups with good GHL may have more confidence in their own health, could actively deal with public health emergencies, and have less psychological burden. Previous studies found that people showing sufficient health literacy were less likely to suffer from psychological problems, such as anxiety, depression, and sleeping disorders [25, 26]. As a result, this study assumed that:

**H5**: *GHL negatively affects CRTPR*.

Another consideration was that the more sufficient CRIRA was, the more likely it was to obtain more positive information, and the less likely the psychological burden in the face of the epidemic will be. Researchers also revealed that high satisfaction with health information, specific up-to-date and accurate health information were significantly associated with lower stress, anxiety, and depression [27, 28]. Accordingly, we supposed that:

**H6**: *CRIRA negatively affects CRTPR*.

#### Hypotheses on indirect effects among different dimensions

In addition to the above direct effects, there may also be some indirect effects among the four dimensions. Therefore, five more hypotheses were proposed as below:

**H7**: *CRHL mediates the relationship between GHL and CRIRA*.

**H8**: *CRIRA mediates the relationship between CRHL and CRTPR*.

**H9**: *CRHL mediates the relationship between GHL and CRTPR*.

**H10**: *CRIRA mediates the relationship between GHL and CRTPR*.

**H11**: *CRHL and CRIRA mediate the relationship between GHL and CRTPR*.

#### Validation of hypotheses

This study adopted the structural equation model (SEM) analysis to quantify the aforementioned relationships among different dimensions. The estimation of SEM relied on the multivariate normal distribution of the data. The item parcel technology could be used to convert categorical scales questions of the questionnaire into continuous variables and transformed their distributions close to the normal ones. Parcels were formed by summing or averaging scores on two or more indicators, which were proven to be more continuous and normally distributed than the individual items [29–35]. Therefore, this study adopted the transformation method and process of parceling proposed by Cattell et al. [29, 30].

The quality of the measurement models was analyzed for their reliability and validity. Cronbach’s alpha and composite reliability were used to evaluate the reliability. The validity testing consisted of convergent validity and discrimination validity. The results showed that the design of measurement models was effective and reasonable, indicating that further structural model fitting analysis could be carried out. More details were shown in Additional file 1.

As a consequence, we incorporated the statistically significant demographic variables obtained from the logistic models as the control variables into the theoretical SEM, and then deleted those demographic variables with insignificant factor load in SEM. In addition, the correlations between the residual terms of SEM were adjusted to improve the fit of the model.

The maximum likelihood method was used to estimate the parameters of SEM. The univariate normal distributions (− 1.438 ≤ skewness ≤ 1.480, − 0.919 ≤ kurtosis ≤ 3.047) and multivariate normal distribution (Mardia’s kurtosis = 45.622) were tested separately. Due to the lack of multivariate normality, the Bollen-Stine bootstrap *p* procedure (performed 5000 times) was used to correct for fit statistic bias [36–40]. The following parameters were used to assess model fitness: relative chi-square (*χ*^2^/*df*), goodness of fit index (GFI), adjust goodness of fit index (AGFI), normed fit index (NFI), Tucker-Lewis index (TLI), incremental fit index (IFI), related fit index (RFI), comparative fit index (CFI), and root mean square approximation error (RMSEA).

Since the bootstrapping method was more powerful than the classical Sobel test and causal steps approach in testing mediating variables effects [41, 42], we adopted the bootstrapping (performed 5000 times) method to analyze the mediating effects of CRHL and CRIRA.

### Estimating the moderating effects of the regional characteristics

It was necessary to pay attention to whether some important regional characteristic variables played significant moderating roles in the relationships among different dimensions. The regional characteristic variables concerned in this study, namely the mediating roles, mainly included the effectiveness of government prevention and control as well as the ethnicity. The effectiveness of government prevention and control of the epidemic may affect people’s use of the media, the acquisition of COVID-19 related information, and even the level of residents’ CRHL. The public’s evaluation of this effectiveness reflects the degree of recognition of government work from a personal perspective. In addition, the current epidemiological data on COVID-19 suggested that minority groups may be more susceptible to COVID-19 infections [43]. To this end, we proposed the last two hypotheses:

**H12**: *The moderating variables moderate the relationships among GHL, CRHL, CRIRA, and CRTPR*.

**H13**: *The moderating variables moderate the relationships among GHL, CRHL, CRIRA, and CRTPR via CRHL and CRIRA*.

To be specific, we constructed moderation models and moderated mediation models to explore the moderating roles of regional characteristic variables.

All the above statistical tests were conducted at the statistically significant level of 0.05 and the statistical analyses were conducted by using SPSS 22.0 (IBM Corp, Armonk, New York, USA) and Amos 26.0 (IBM Corp, Armonk, New York, USA) software.

## Results

### Basic characteristics of participants

A total of 1789 questionnaires were collected with a response rate of 85.2%. After eliminating 118 responses with missing values and outliers, 1671 valid questionnaires were obtained with an effective rate of 93.4%. The demographic characteristics of participants were shown in Table 2. The proportions across categories of gender and age kept balanced. Most of the participants were Han (47.3%) and Yi nationality (40.7%). The majority (86.7%) were married. More than half of the participants (60.1%) received no higher education than primary school, and only 6.5% received college education or above. Of the 1671 respondents, 84.0% (*n* = 1404) were farmers and the majority (83.0%) had an annual income of ≤ CNY 10,000.

**Table 2:** Demographic characteristics and their influence on each dimension estimated by logistic regression

Variable and category	*n* (%)	*OR* (95% *CI*)			
		GHL	CRHL	CRIRA	CRTPR
Gender			/	/	/
Male	738 (44.2)				
Female	933 (55.8)	0.686^*^ (0.539‒0.872)			
Age, years					
≤ 19	49 (2.9)				
20–29	314 (18.8)	2.020 (0.801‒5.479)	2.629^*^ (1.100‒6.878)	4.833^*^ (1.935‒14.757)	0.848 (0.392‒1.765)
30–39	385 (23.0)	2.428 (0.948‒6.673)	2.644^*^ (1.099‒6.946)	4.901^*^ (1.949‒15.047)	1.033 (0.479‒2.138)
40–49	406 (24.3)	1.544 (0.598‒4.266)	2.916^*^ (1.214‒7.657)	3.984^*^ (1.583‒12.234)	0.502 (0.234‒1.034)
50–59	332 (19.9)	1.060 (0.404‒2.965)	3.280^*^ (1.355‒8.671)	3.619^*^ (1.425‒11.184)	0.516 (0.238‒1.075)
60–69	185 (11.1)	1.020 (0.374‒2.957)	4.722^*^ (1.901‒12.760)	4.555^*^ (1.753‒14.297)	0.289^*^ (0.127‒0.634)
Ethnicity			/		
Han	791 (47.3)				
Yi	680 (40.7)	0.308^*^ (0.230‒0.411)		0.603^*^ (0.480‒0.757)	0.601^*^ (0.451‒0.803)
Tibetan	88 (5.3)	0.609^*^ (0.369‒0.997)		1.006 (0.638‒1.578)	1.535 (0.912‒2.611)
Miao	68 (4.1)	1.550 (0.883‒2.777)		0.822 (0.487‒1.368)	0.631 (0.357‒1.110)
Hui	22 (1.3)	2.886 (0.900‒12.913)		1.132 (0.464‒2.741)	1.168 (0.429‒3.742)
Other	22 (1.3)	0.709 (0.271‒1.870)		2.470^*^ (1.020‒6.581)	1.213 (0.440‒3.471)
Marital status			/	/	/
Unmarried	154 (9.2)				
Married	1,449 (86.7)	2.013^*^ (1.215‒3.355)			
Separated	2 (0.1)	0.455 (0.011‒17.363)			
Divorced	18 (1.1)	8.111^*^ (1.837‒58.736)			
Widowed	48 (2.9)	2.280 (0.908‒5.596)			
Educational level			/	/	
Illiterate or barely literate	575 (34.4)				
Primary school	429 (25.7)	1.546^*^ (1.146‒2.087)			2.423^*^ (1.803‒3.266)
Junior high school	406 (24.3)	2.915^*^ (2.089‒4.078)			5.072^*^ (3.629‒7.123)
Senior high school / vocational high school / technical secondary school	152 (9.1)	9.141^*^ (5.007‒17.526)			6.956^*^ (4.198‒11.810)
Junior college	90 (5.4)	12.396^*^ (5.060‒34.427)			9.196^*^ (4.690‒19.543)
Undergraduate or above	19 (1.1)	3.689 (0.898‒19.217)			5.420^*^ (1.662‒24.485)
Occupation					/
Teacher	18 (1.1)				
Medical staff	47 (2.8)	0.768 (0.131‒3.742)	11.338^*^ (3.183‒44.187)	2.241 (0.728‒7.129)	
Personnel of other institutions	62 (3.7)	0.881 (0.157‒4.010)	3.899^*^ (1.259‒12.669)	1.663 (0.565‒5.036)	
Student	29 (1.7)	17.367^*^ (1.580‒424.614)	9.328^*^ (2.176‒44.701)	7.518^*^ (1.756‒36.609)	
Farmer	1,404 (84.0)	0.515 (0.106‒1.894)	3.093^*^ (1.146‒8.784)	0.842 (0.325‒2.256)	
Worker	35 (2.1)	0.398 (0.070‒1.812)	0.906 (0.246‒3.344)	0.566 (0.167‒1.903)	
Other	76 (4.6)	1.031 (0.191‒4.445)	3.057^*^ (1.022‒9.586)	1.274 (0.445‒3.744)	
Annual income, CNY			/	/	/
≤ 5000	784 (46.9)				
5000–10,000	603 (36.1)	1.533^*^ (1.191‒1.976)			
10,000–15,000	127 (7.6)	1.277 (0.817‒1.996)			
15,000–20,000	82 (4.9)	1.511 (0.878‒2.629)			
> 20,000	75 (4.5)	2.146^*^ (1.135‒4.179)			
GHL		–	6.949^*^ (5.535‒8.764)	/	2.492^*^ (1.904‒3.270)
CRHL		–	–	1.171 (0.952‒1.440)	0.763^*^ (0.588‒0.986)
CRIRA		–	–	–	1.403^*^ (1.109‒1.775)

– Not included in the model. / Variables not selected by stepwise regression method. *CRHL* COVID-19 related health literacy, *GHL* general health literacy, *CRIRA* COVID-19 related information report acquisition, *CRTPR* COVID-19 related tense psychological reactions. *P < 0.05

As shown in Table 3, the level of CRHL reached 52.5%, with 16.3% in basic knowledge of COVID-19, 62.8% in prevention behavior knowledge of COVID-19, and 63.1% in protection skills of COVID-19. A total of 94.5% of the respondents agreed or strongly agreed with the sufficiency of all aspects of their CRIRA. Only 14.0% of the respondents had obvious tension and reaction to the COVID-19. However, the level of GHL was only 10.7%.

**Table 3:** The regional level of the four dimensions of the questionnaire

Variable	Level
CRHL	52.5%
Basic knowledge of COVID-19	16.3%
Prevention behavior knowledge of COVID-19	62.8%
Protection skills of COVID-19	63.1%
GHL	10.7%
Scientific health view	18.3%
Infectious disease prevention and control literacy	12.5%
Chronic disease prevention and control literacy	12.1%
Basic medical literacy	10.3%
Health information literacy	16.4%
CRIRA	Disagree (0.0%) Disagree somewhat (0.2%) Generally (5.3%) Agree (53.6%) Agree strongly (40.9%)
Information source authority	
Information reporting adequacy	
Information expression intelligibility	
Information content practicability	
CRTPR	Not nervous (49.2%) A little nervous (36.7%) Nervous relatively (10.2%) Nervous (2.4%) Nervous strongly (1.4%)

*CRHL* COVID-19 related health literacy, *GHL* general health literacy, *CRIRA* COVID-19 related information report acquisition, *CRTPR* COVID-19 related tense psychological reactions

### Influence of demographic characteristics

As shown in Table 2, the results of logistic models showed that most of the demographic characteristics with statistical significance were poverty-oriented factors. Age and occupation were significantly associated with CRHL. The older the age was, the higher the CRHL level was (*OR*s > 1). Age, ethnicity, and occupation were significantly associated with CRIRA. Compared with the low-age reference group (≤ 19), other age groups had more adequate CRIRA (*OR*s > 1). Compared with the Han nationality, the sufficiency of the CRIRA of the Yi nationality was weaker (*OR* = 0.603, 95% *CI*: 0.480‒0.757). Age, ethnicity, and educational level were significantly associated with CRTPR. Elderly people aged 60 and above were less likely to show nervous reactions in the face of the epidemic (*OR* = 0.289, 95% *CI*: 0.127‒0.634). The Yi nationality was less prone to tension than the Han nationality (*OR* = 0.601, 95% *CI*: 0.451‒0.803). Overall, the higher the level of education was, the easier it was to be nervous (*OR*s > 1). For the GHL, other demographic variables except age were statistically significant. Women had lower GHL level than men (*OR* = 0.686, 95% *CI*: 0.539‒0.872). The GHL levels of Yi (*OR* = 0.308, 95% *CI*: 0.230‒0.411) and Tibetan (*OR* = 0.609, 95% *CI*: 0.369‒0.997) minorities were lower than that of Han nationality. The lower the education level and income level were, the more limited the GHL level was (*OR*s > 1).

**Fig. 2:**
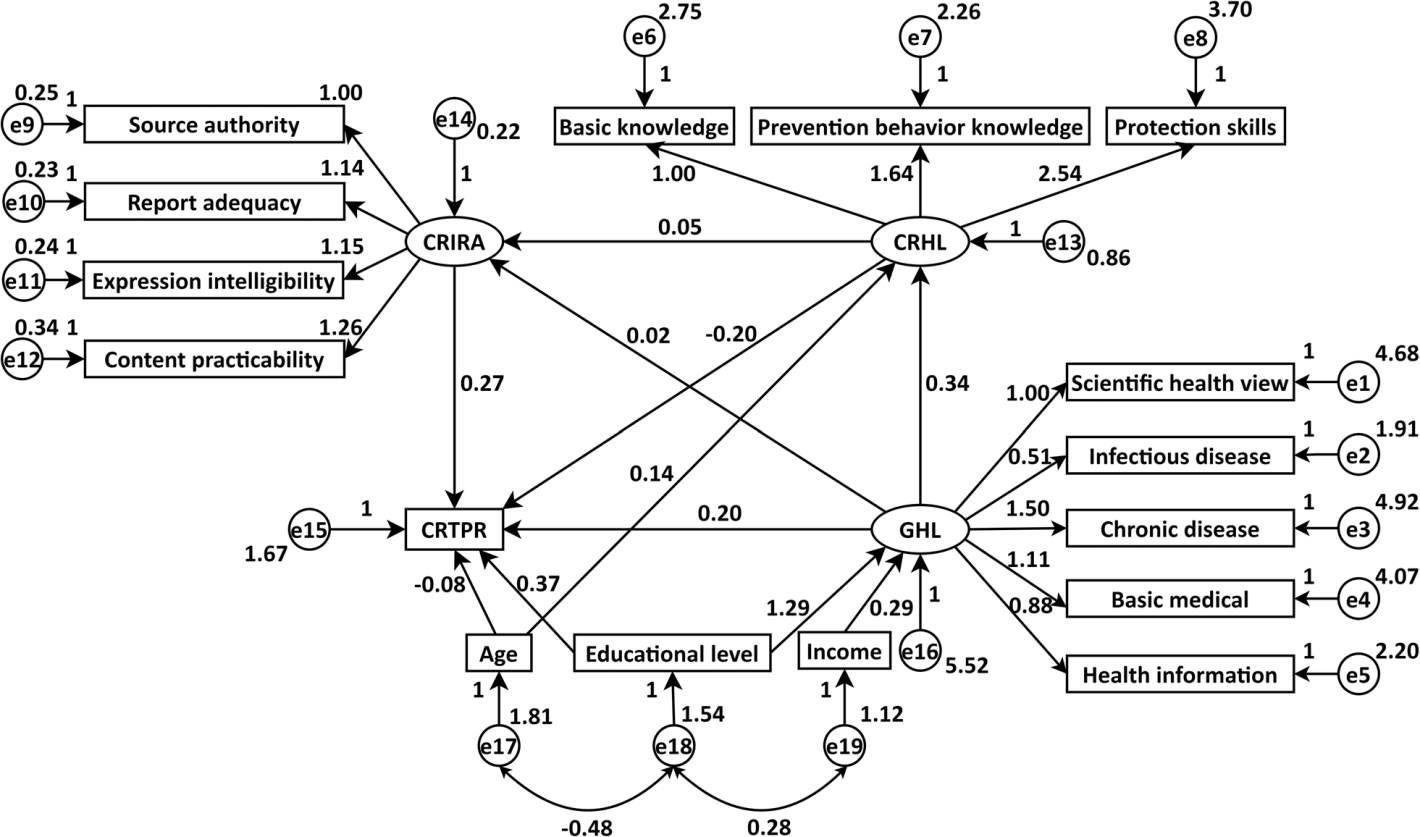
The unstandardized estimation results of the structural equation model. *CRHL* COVID-19 related health literacy, *GHL* general health literacy, *CRIRA* COVID-19 related information report acquisition, *CRTPR* COVID-19 related tense psychological reactions

Next, we incorporated the aforementioned statistically significant demographic characteristics as control variables into the theoretical SEM, deleted the variables with insignificant factor load, and made appropriate adjustment. As shown in Fig. 2, the results showed that age (*β* = 0.136, *P* < 0.001) had a significant impact on CRHL, age (*β* = − 0.080, *P* = 0.002) and educational level (*β* = 0.366, *P* < 0.001) had significant impacts on CRTPR, and income (*β* = 0.286, *P* < 0.001) and educational level (*β* = 1.292, *P* < 0.001) had significant impacts on GHL. However, no demographic variable in the SEM had a statistically significant association with CRIRA.

### Relationships among different dimensions

Table 4 presented the assessment results of SEM fitting. It showed that all the raw values of indices (column 2), except for those of *χ*^2^/*df* and RMSEA, were acceptable compared with the criteria (column 4). However, after adopting the Bollen-Stine bootstrap to correct for the fit statistic bias, all the fitting indices were ideal (column 3).

**Table 4:** Results of the overall model fitting

Index	Raw value	Bollen-Stine bootstrap	Criterion
*χ*^2^	502.9667	98.5833	–
*df*	95	95	–
*χ*^2^/*df*	5.2944	1.0377	< 3
GFI	0.9630	0.9923	> 0.9
AGFI	0.9470	0.9864	> 0.9
NFI	0.9606	0.9923	> 0.9
TLI	0.9593	0.9996	> 0.9
IFI	0.9678	0.9997	> 0.9
RFI	0.9503	0.9903	> 0.9
CFI	0.9678	0.9997	> 0.9
RMSEA	0.0507	0.0048	< 0.05

– Means not applicable. *GFI* goodness of fit index, *AGFI* adjust goodness of fit index, *NFI* normed fit index, *TLI* Tucker-Lewis index, *IFI* incremental fit index, *RFI* related fit index, *CFI* comparative fit index, *RMSEA* root mean square approximation error

The unstandardized path coefficients of SEM were shown in Table 5. All the direct and indirect effects proposed were statistically significant, and such findings supported most of the proposed hypotheses in Methods section, albeit that for the H5 and H6, the estimated results showed positive effects, which were contrary to the proposed hypotheses. Considering the status quo of Liangshan Yi Autonomous Prefecture, the positive effects of GHL and CRIRA on CRTPR were most likely due to the fact that the explosive growth of COVID-19 related information after its outbreak formed sharp contrast to the previous problem of information deficiency in local area, which consequently led some residents to the excessive risk perception and tension.

**Table 5:** Unstandardized direct, indirect and total effects of the four dimensions in the structural equation model

Relationship	Point estimate (*β*)	Product of coefficient		Bootstrapping			
				Percentile 95% *CI*		Bias-corrected percentile 95% *CI*	
		*SE*	*Z*	Lower	Upper	Lower	Upper
Direct effect							
H1: *CRHL to CRTPR*	− 0.198	0.042	− 4.714	− 0.283	− 0.119	− 0.282	− 0.118
H2: *CRHL to CRIRA*	0.052	0.018	2.889	0.019	0.087	0.019	0.089
H3: *GHL to CRHL*	0.336	0.016	21.000	0.304	0.368	0.303	0.368
H4: *GHL to CRIRA*	0.025	0.007	3.571	0.011	0.039	0.011	0.039
H5: *GHL to CRTPR*	0.195	0.025	7.800	0.148	0.245	0.147	0.244
H6: *CRIRA to CRTPR*	0.266	0.080	3.325	0.109	0.425	0.115	0.431
Indirect effect:							
H7: *GHL to CRHL to CRIRA*	0.018	0.006	3.000	0.006	0.029	0.007	0.030
H8: *CRHL to CRIRA to CRTPR*	0.014	0.007	2.000	0.003	0.029	0.004	0.032
H9: *GHL to CRHL to CRTPR*	− 0.066	0.015	− 4.400	− 0.097	− 0.040	− 0.096	− 0.039
H10: *GHL to CRIRA to CRTPR*	0.007	0.003	2.333	0.002	0.013	0.003	0.014
H11: *GHL to CRHL to CRIRA to CRTPR*	0.005	0.002	2.500	0.001	0.010	0.001	0.010
Total effect:							
*CRHL to CRTPR*	− 0.184	0.042	− 4.381	− 0.268	− 0.106	− 0.266	− 0.105
*CRHL to CRIRA*	0.052	0.018	2.889	0.019	0.087	0.019	0.089
*GHL to CRHL*	0.336	0.016	21.000	0.304	0.368	0.303	0.368
*GHL to CRIRA*	0.042	0.005	8.400	0.033	0.052	0.034	0.052
*GHL to CRTPR*	0.140	0.016	8.750	0.109	0.173	0.109	0.172
*CRIRA to CRTPR*	0.266	0.080	3.325	0.109	0.425	0.115	0.431
Contrast:							
H9 vs H10	− 0.073	0.015	− 4.867	− 0.104	− 0.045	− 0.104	− 0.045
H9 vs H11	− 0.071	0.015	− 4.733	− 0.102	− 0.044	− 0.102	− 0.043
H10 vs H11	0.002	0.003	0.667	− 0.005	0.009	− 0.004	0.010

*CRHL* COVID-19 related health literacy, *GHL* general health literacy, *CRIRA* COVID-19 related information report acquisition, *CRTPR* COVID-19 related tense psychological reactions

Table 5 displayed the relationships among the four dimensions, where CRHL was the key part. There was a significant negative relationship between CRHL and CRTPR (*β* = − 0.198), but a positive relationship between GHL and CRTPR (*β* = 0.195). Meanwhile, the CRHL had a positive effect on CRIRA (*β* = 0.052), and CRIRA had a positive effect on CRTPR (*β* = 0.266). The higher the GHL level was, the higher the CRHL level was (*β* = 0.336).

From the results of mediating effects analysis, both the paths *GHL to CRHL to CRIRA to CRTPR* (*β* = 0.005) and *GHL to CRIRA to CRTPR* (*β* = 0.007) had positive effects, and it was found that their effect difference was not statistically significant after contrasts (H10 vs H11). However, the path *GHL to CRHL to CRTPR* (*β* = − 0.066) had a negative effect, which was mainly due to the negative effect of CRHL on CRTPR. This specific indirect effect accounted for 84.6% of the total indirect effect, which had a great impact on the effect of GHL on CRTPR.

### Moderation and moderated mediation effects of regional characteristics

**Fig. 3:**
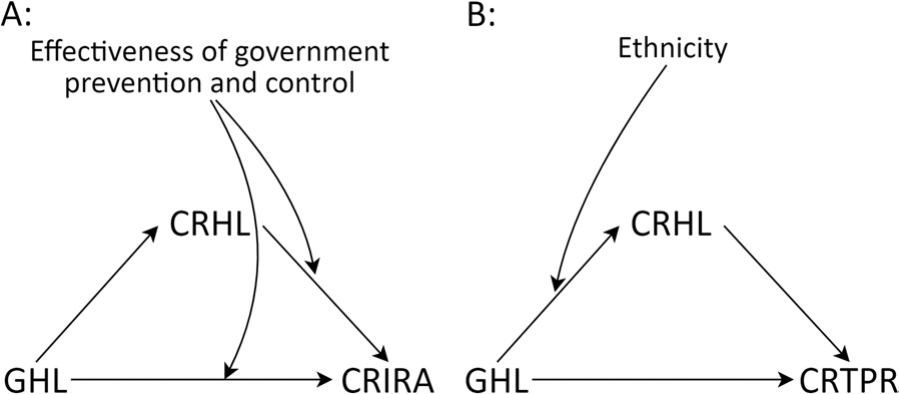
The basic moderation models and moderated mediation model. **A** effectiveness of government prevention and control as moderation variable. **B** ethnicity as moderation variable. *CRHL* COVID-19 related health literacy, *GHL* general health literacy, *CRIRA* COVID-19 related information report acquisition, *CRTPR* COVID-19 related tense psychological reactions

**Fig. 4:**
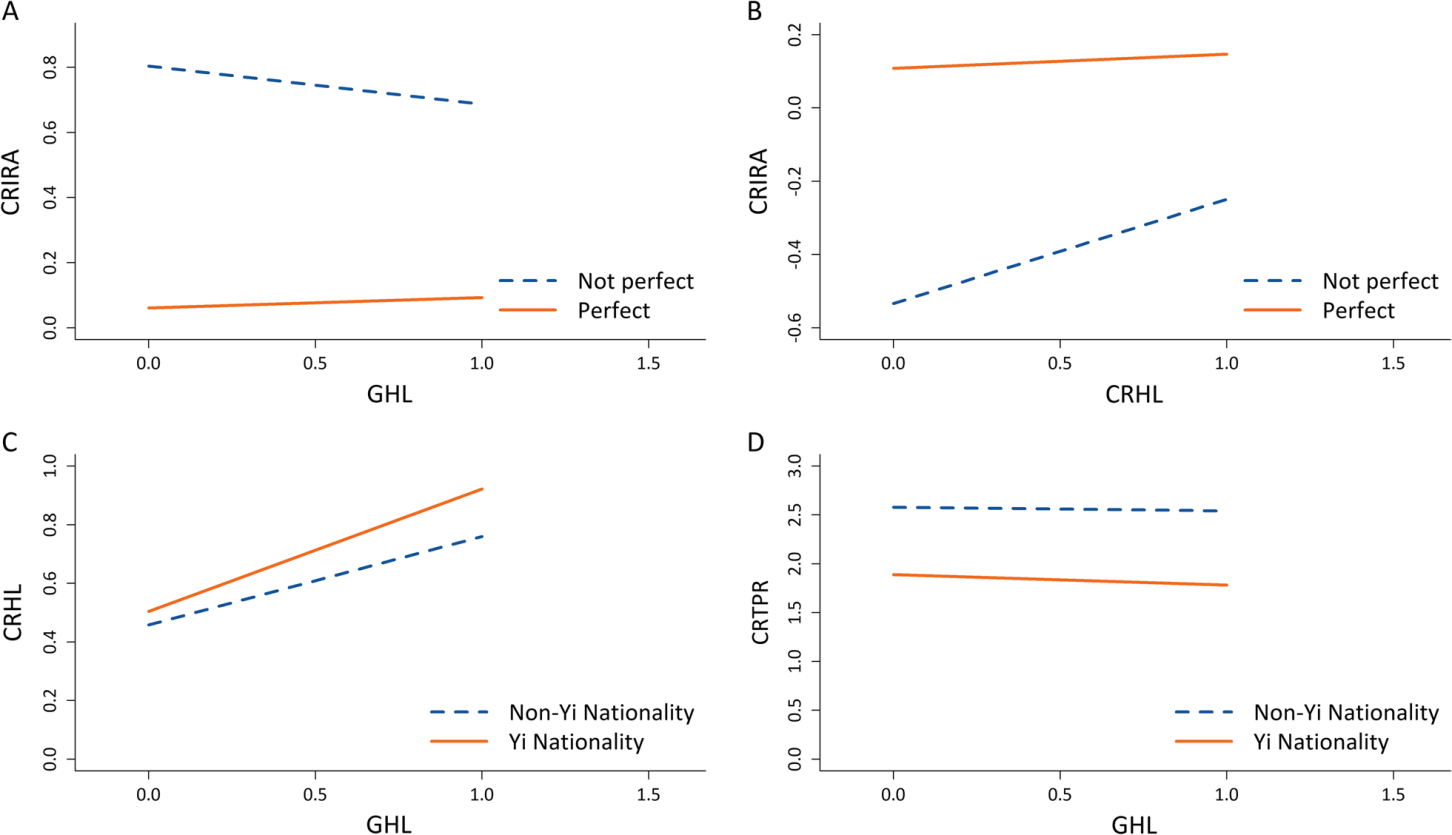
The moderating effects of moderation variables on the paths. **A** Effectiveness of government prevention and control moderated the *GHL to CRIRA* path. **B** Effectiveness of government prevention and control moderated the *CRHL to CRIRA* path. **C** Ethnicity moderated the *GHL to CRHL* path. **D** Ethnicity moderated the mediation effect of CRHL on the *GHL to CRTPR* path. *CRHL* COVID-19 related health literacy, *GHL* general health literacy, *CRIRA* COVID-19 related information report acquisition, *CRTPR* COVID-19 related tense psychological reactions

The basic information of moderating variables was presented in Table 6. Figure 3 was the schematic diagrams of the basic moderation models and moderated mediation model. Figure 4 illustrated that the moderating effects of moderation variables on the paths, where the slope represented the strength of association between abscissa and ordinate variables.

**Table 6:** Statistical description of moderating variables

Variable and category	*n* (%)
Effectiveness of government prevention and control	
Not perfect	67 (4.0)
Perfect	1604 (96.0)
Ethnicity	
Yi nationality	680 (40.7)
Non-Yi nationality	991 (59.3)

As shown in Table 7, the effectiveness of government prevention and control significantly moderated the effect of CRHL on CRIRA (*χ*^2^ = 6.433, *P* = 0.011), as well as the effect of GHL on CRIRA (*χ*^2^ = 12.105, *P* = 0.001). From Fig. 4A and B, for those participants who considered the government’s prevention and control was not perfect, the CRHL was positively associated with CRIRA (*β* = 0.285), but GHL was negatively associated with CRIRA (*β* = − 0.117). Meanwhile, as shown in Fig. 4B, the slope of the group who held the government’s prevention and control was not perfect was considerably larger than that of the other group.

**Table 7:** The test significance of moderating effects

Path	Effectiveness of government prevention and control		Ethnicity	
	*χ*^2^ (*df*)	*P*	*Χ*^2^ (*df*)	*P*
H1: *CRHL to CRTPR*	0.230 (1)	0.631	2.084 (1)	0.149
H2: *CRHL to CRIRA*	6.433 (1)	0.011	1.707 (1)	0.191
H3: *GHL to CRHL*	1.586 (1)	0.208	10.641 (1)	0.001
H4: *GHL to CRIRA*	12.105 (1)	0.001	2.673 (1)	0.102
H5: *GHL to CRTPR*	0.487 (1)	0.485	1.184 (1)	0.276
H6: *CRIRA to CRTPR*	0.070 (1)	0.792	0.081 (1)	0.775

*CRHL* COVID-19 related health literacy, *GHL* general health literacy, *CRIRA* COVID-19 related information report acquisition, *CRTPR* COVID-19 related tense psychological reactions

Ethnicity significantly moderated the effect of GHL on CRHL (*χ*^2^ = 10.641, *P* = 0.001). Meanwhile, as illustrated in the moderated mediation effect results of Table 8, ethnicity moderated the mediating effect of CRHL on the relationship between GHL and CRTPR. As shown in Fig. 4C and D, the slope of the Yi nationality was considerably larger than that of the non-Yi nationality. It could be explained that the CRHL promotion and CRTPR mitigation work carried out by the local government have generally achieved good results, especially for the Yi nationality.

**Table 8:** Path coefficients of *GHL to CRHL to CRTPR* in terms of ethnicity moderating the mediation effect of CRHL

Moderate	Point estimate	Product of coefficient		Bootstrapping			
				Percentile 95% *CI*		Bias-corrected percentile 95% *CI*	
		*SE*	*Z*	Lower	Upper	Lower	Upper
Non-Yi nationality	− 0.039	0.020	− 1.950	− 0.080	− 0.002	− 0.079	− 0.001
Yi nationality	− 0.108	0.025	− 4.320	− 0.161	− 0.063	− 0.159	− 0.062
Non-Yi nationality vs Yi nationality	0.069	0.032	2.156	0.008	0.134	0.008	0.134

*GHL* general health literacy, *CRHL* COVID-19 related health literacy, *CRTPR* COVID-19 related tense psychological reactions

## Discussion

After the outbreak of COVID-19, the local government of Liangshan Yi Autonomous Prefecture established the epidemic information release mechanism and made timely and prompt dissemination about the prevention and control regulations [11], which effectively promoted the CRHL. However, the GHL level used to remain very low due to the weak health service capacity and long-term low socio-economic development level in Liangshan Yi Autonomous Prefecture. This study verified that the theoretical framework based on the integration of previous researches from different regions was basically suitable for impoverished regions with only slight exceptions. On this basis, combining the theoretical results and the demographic and regional characteristics of impoverished regions, this study put forward the following suggestions.

The mediating effect of CRHL between GHL and CRTPR suggested that reducing public anxiety during the COVID-19 epidemic could be carried out in two ways. First, from the perspective of emergency response, the increase of CRTPR caused by the difficulties of the public to understand professional health information could be alleviated by enhancing the CRHL. Therefore, the government should efficiently provide updated information about the COVID-19 for the public to avoid social panic. In addition, from the perspective of long-term prevention and control of public health emergencies, since the GHL is the crucial cornerstone of CRHL, it indicated that the construction of a long-term mechanism for GHL improvement in impoverished regions cannot be ignored. Only by ensuring long-term, sustained, and effective health education in impoverished regions could the health literacy of residents be truly improved, thereby further enhancing their abilities to cope with public health emergencies.

In the course of health education work, this study also suggested some vulnerable groups should deserve much attention. For example, long-term GHL education could be focused on the vulnerable groups with low income and education levels. For low-income individuals, the medical workers appointed by the government could cultivate a person with relatively high health literacy for each low-income family by means of group assistance with the family as the unit. Meanwhile, the employment training and public welfare jobs could also be provided for low-income people, thereby improving their affordability to maintain the healthy level. For low-educated individuals, the first suggestion was that family physicians could jointly carry out health education with local religious and cultural representatives, which can give full play to the role in promoting health. Secondly, profound prevention and control knowledge could be made into easy-to-understand science materials close to regional characteristics, such as audio and wall charts. In addition, experts can also be invited to conduct health lectures combined with specific cases and guide on-site drills to promote the improvement of GHL.

In addition to education and income factors, special attention should also be paid to the youth in the impoverished regions. Because their outlook on life and values have not yet been fully formed, they lack discrimination against rumors and are prone to be impulsive when facing public health emergencies. Therefore, in addition to the local efforts to promote COVID-19 knowledge through new media such as the official website and WeChat public accounts, we highly recommended consolidating school education and peer education for the youth, as well as the connection with their family members. Through the above measures, the CRHL of young people could be improved comprehensively and their anxiety could also be further alleviated.

Besides the health education work itself, the government should also implement timely assessment of the work effects for further improvement. This study showed although the vast majority (96.0%) were satisfied with the government’s epidemic prevention and control work, there were also a small number of people who thought that the government’s prevention and control was not perfect. Furthermore, the dissatisfaction mainly came from people with relatively high education and income in society. Since the key groups for the impoverished regions in the emergency state of the outbreak were those with low income and education levels, it was understandable that health education work in the early-stage of COVID-19 could hardly take care of everyone’s interest. However, with the in-depth development of the scientific prevention and control strategy of COVID-19, the government should consider conducting timely assessment of work effects. It is also necessary to listen to the feedback of different groups, and adjust the prevention and control strategies dynamically according to the epidemic, so as to improve the public’s satisfaction and confidence in the government’s efforts on epidemic prevention and control, and steadily realize the normalized management of the epidemic.

Overall, this study made both theoretical and practical contributions to improving the COVID-19 health education strategy in impoverished regions. From the theoretical perspective, a set of hypotheses framework was specifically proposed for impoverished regions, and its validity was also checked by our pilot study in Liangshan Yi Autonomous Prefecture. Besides, in terms of practice, this study put forward specific suggestions on how to improve COVID-19 health education strategies in impoverished regions, involving *implementation methods*, *key groups* and *effect evaluation*. These suggestions have been partially adopted by the Liangshan Prefecture Center for Disease Control and Prevention, and some preliminary work has already been initiated.

However, it should also be acknowledged that there were still limitations in our study. For example, self-reported measures may lead to bias and inaccurate estimation of measured variables with the interference of subjective factors. Besides, this was a cross-sectional design study. It is highly expected that future work could be carried out on the longitudinal study of the dynamic effects of health education work.

## Conclusions

Although it was difficult to raise the public CRHL in impoverished regions, this study proposed and verified the theoretical framework of the relationships among several dimensions under the COVID-19 health education framework. Specific suggestions on how to improve COVID-19 health education strategies in impoverished regions were put forward. These suggestions would also provide ideas for the popularization of health literacy and the development of health education of other public health emergencies in impoverished regions in the future.

## Supplementary Information

**Additional file 1.** The reliability and validity analysis of measurement models.

## Data Availability

The data that support the findings of this study are available from Liangshan Prefecture Center for Disease Control and Prevention but restrictions apply to the availability of these data, which were used under license for the current study, and so are not publicly available. Data are however available from the authors upon reasonable request and with permission of Liangshan Prefecture Center for Disease Control and Prevention.

## Abbreviations

COVID-19: Coronavirus disease 2019
CRHL: COVID-19 related health literacy
CRTPR: COVID-19 related tense psychological reactions
CRIRA: COVID-19 related information report acquisition
GHL: General health literacy
PPS: Probability proportionate to size sampling
SEM: Structural equation model
GFI: Goodness of fit index
AGFI: Adjust goodness of fit index
NFI: Normed fit index
TLI: Tucker-Lewis index
IFI: Incremental fit index
RFI: Related fit index
CFI: Comparative fit index
RMSEA: Root mean square approximation error

## References

[1] UNICEF Sudan. Combatting myths and misinformation at Sudan’s COVID-19 Hotline Call Centre. 2020. https://www.unicef.org/sudan/stories/combatting-myths-and-misinformation-sudans-covid-19-hotline-call-centre. Accessed 18 Nov 2021.

[2] CGTN Africa. Misconceptions on COVID-19 persists in Kenya’s rural areas. 2020. https://africa.cgtn.com/2020/08/24/misconceptions-on-covid-19-persists-in-kenyas-rural-areas/. Accessed 18 Nov 2021.

[3] Patil U, Kostareva U, Hadley M, Manganello JA, Okan O, Dadaczynski K (2021). Health literacy, digital health literacy, and COVID-19 pandemic attitudes and behaviors in US college students: implications for interventions. Int J Environ Res Public Health.

[4] Okan O, Bollweg TM, Berens EM, Hurrelmann K, Bauer U, Schaeffer D (2020). Coronavirus-related health literacy: a cross-sectional study in adults during the COVID-19 infodemic in Germany. Int J Environ Res Public Health.

[5] Isautier JM, Copp T, Ayre J, Cvejic E, Meyerowitz-Katz G, Batcup C (2020). People’s experiences and satisfaction with telehealth during the COVID-19 pandemic in Australia: cross-sectional survey study. J Med Internet Res.

[6] Li S, Cui G, Kaminga AC, Cheng S, Xu H (2021). Associations between health literacy, eHealth literacy, and COVID-19-related health behaviors among Chinese college students: cross-sectional online study. J Med Internet Res.

[7] Nguyen HT, Do BN, Pham KM, Kim GB, Dam HTB, Nguyen TT (2020). Fear of COVID-19 scale-associations of its scores with health literacy and health-related behaviors among medical students. Int J Environ Res Public Health.

[8] Do BN, Tran TV, Phan DT, Nguyen HC, Nguyen TTP, Nguyen HC (2020). Health literacy, eHealth literacy, adherence to infection prevention and control procedures, lifestyle changes, and suspected COVID-19 symptoms among health care workers during lockdown: online survey. J Med Internet Res.

[9] Duong TV, Lin CY, Chen SC, Huang YK, Okan O, Dadaczynski K (2021). Oxford COVID-19 vaccine hesitancy in school principals: impacts of gender, well-being, and coronavirus-related health literacy. Vaccines.

[10] Duong MC, Nguyen HT, Duong BT, Vu MT (2021). The levels of COVID-19 related health literacy among university students in Vietnam. Infect Chemother.

[11] Liao RJ, Ji-Ke CN, Zhang T, Liao Q, Li L, Zhu TY (2020). Coronavirus disease 2019 epidemic in impoverished area: Liangshan Yi autonomous prefecture as an example. Infect Dis Poverty.

[12] Department of Publicity, National Health Commission of the People’s Republic of China. Chinese health literacy monitoring. 2017. http://www.nhc.gov.cn/xcs/hyzl/201704/f23889d798054889a8ff028ee0a00094.shtml. Accessed 18 Nov 2021.

[13] Kish L (1949). A procedure for objective respondent selection within the household. J Am Stat Assoc.

[14] Wang H, Tang J, Wu M, Wang X, Zhang T (2022). Application of machine learning missing data imputation techniques in clinical decision making: taking the discharge assessment of patients with spontaneous supratentorial intracerebral hemorrhage as an example. BMC Med Inform Decis Mak.

[15] Fernández RS, Crivelli L, Guimet NM, Allegri RF, Pedreira ME (2020). Psychological distress associated with COVID-19 quarantine: latent profile analysis, outcome prediction and mediation analysis. J Affect Disord.

[16] Nutbeam D, Lloyd JE (2021). Understanding and responding to health literacy as a social determinant of health. Annu Rev Public Health.

[17] Paasche-Orlow MK, Parker RM, Gazmararian JA, Nielsen-Bohlman LT, Rudd RR (2005). The prevalence of limited health literacy. J Gen Intern Med.

[18] Qin L, Xu H (2016). A cross-sectional study of the effect of health literacy on diabetes prevention and control among elderly individuals with prediabetes in rural China. BMJ Open.

[19] Hossain MA, Jahid MIK, Hossain KMA, Walton LM, Uddin Z, Haque MO (2020). Knowledge, attitudes, and fear of COVID-19 during the rapid rise period in Bangladesh. PLoS One.

[20] Wu M, Han H, Lin T, Chen M, Wu J, Du X (2020). Prevalence and risk factors of mental distress in China during the outbreak of COVID-19: a national cross-sectional survey. Brain Behav.

[21] Lee JJ, Kang KA, Wang MP, Zhao SZ, Wong JYH, O'Connor S (2020). Associations between COVID-19 misinformation exposure and belief with COVID-19 knowledge and preventive behaviors: cross-sectional online study. J Med Internet Res.

[22] An L, Bacon E, Hawley S, Yang P, Russell D, Huffman S (2021). Relationship between coronavirus-related eHealth literacy and COVID-19 knowledge, attitudes, and practices among US adults: web-based survey study. J Med Internet Res.

[23] Luengo-Oroz M, Pham KH, Bullock J, Kirkpatrick R, Luccioni A, Rubel S (2020). Artificial intelligence cooperation to support the global response to COVID-19. Nat Mach Intell.

[24] Ashrafi-Rizi H, Kazempour Z (2020). Information typology in coronavirus (COVID-19) crisis; a commentary. Arch Acad Emerg Med..

[25] Hermans L, Van den Broucke S, Gisle L, Demarest S, Charafeddine R (2021). Mental health, compliance with measures and health prospects during the COVID-19 epidemic: the role of health literacy. BMC Public Health.

[26] Do BN, Nguyen PA, Pham KM, Nguyen HC, Nguyen MH, Tran CQ (2020). Determinants of health literacy and its associations with health-related behaviors, depression among the older people with and without suspected COVID-19 symptoms: a multi-institutional study. Front Public Health.

[27] Wang C, Pan R, Wan X, Tan Y, Xu L, McIntyre RS (2020). A longitudinal study on the mental health of general population during the COVID-19 epidemic in China. Brain Behav Immun.

[28] Wang C, Pan R, Wan X, Tan Y, Xu L, Ho CS (2020). Immediate psychological responses and associated factors during the initial stage of the 2019 coronavirus disease (COVID-19) epidemic among the general population in China. Int J Environ Res Public Health.

[29] Cattell RB (1956). Validation and intensification of the sixteen personality factor questionnaire. J Clin Psychol.

[30] Cattell RB (1974). Radial item parcel factoring vs item factoring in defining personality structure in questionnaires: theory and experimental checks. Aust J Psychol.

[31] Bandalos DL, Finney SJ, Marcoulides GA, Schumacker RE (2001). Item parceling issues in structural equation modeling. New developments and techniques in structural equation modeling.

[32] Bandalos DL (2002). The effects of item parceling on goodness-of-fit and parameter estimate bias in structural equation modeling. Struct Equ Modeling.

[33] Nasser F, Takahashi T (2003). The effect of using item parcels on ad hoc goodness-of-fit indexes in confirmatory factor analysis: an example using Sarason’s reactions to tests. Appl Meas Educ.

[34] Hau KT, Marsh HW (2004). The use of item parcels in structural equation modeling: non-normal data and small sample sizes. Br J Math Stat Psychol.

[35] Matsunaga M (2008). Item parceling in structural equation modeling: a primer. Commun Methods Meas.

[36] Gao S, Mokhtarian PL, Johnston RA (2008). Nonnormality of data in structural equation models. Transp Res Rec.

[37] Muthén B, Kaplan D (1985). A comparison of methodologies for the factor analysis of non-normal likert variables. Br J Math Stat Psychol.

[38] Harlow LL: Behavior of some elliptical theory estimators with non-normality data in a covariance structures framework: a Monte Carlo study. https://www.proquest.com/docview/303360947/A880C451A61A4D6EPQ (1985). Accessed 25 Jan 2022.

[39] Bollen KA, Stine RA (1992). Bootstrapping goodness-of-fit measures in structural equation models. Sociol Method Res.

[40] Enders CK (2005). An SAS macro for implementing the modified Bollen-Stine bootstrap for missing data: implementing the bootstrap using existing structural equation modeling software. Struct Equ Modeling.

[41] Hayes AF (2009). Beyond Baron and Kenny: statistical mediation analysis in the new millennium. Commun Monogr.

[42] Williams J, Mackinnon DP (2008). Resampling and distribution of the product methods for testing indirect effects in complex models. Struct Equ Modeling.

[43] Garg S, Kim L, Whitaker M, O'Halloran A, Cummings C, Holstein R (2020). Hospitalization rates and characteristics of patients hospitalized with laboratory-confirmed coronavirus disease 2019-COVID-NET, 14 States, March 1–30, 2020. MMWR Morb Mortal Wkly Rep.

