# Supplementary material for: How to improve the COVID-19 health education strategy in impoverished regions: a pilot study
Source: Infect Dis Poverty. 2022 Mar 29;11:38. doi: 10.1186/s40249-022-00963-3 (PMC8961089; doi:10.1186/s40249-022-00963-3)
Supplement: Supplementary file 1 — Additional file 1. The reliability and validity analysis of measurement models. [file 40249_2022_963_MOESM1_ESM.docx]

**Additional file: The reliability and validity analysis of measurement models.**

The quality of the measurement models can be analyzed for its reliability and validity. Cronbach’s alpha and composite reliability (CR) reflected the reliability of the measurement indicators of the latent variables, with the recommended values higher than 0.7 [1]. The factor load and average variance extraction (AVE) were used to test the convergent validity. The recommended value for the factor load was higher than 0.5 or ideal 0.7, and for AVE was higher than 0.5 [1]. The discrimination validity could be measured by the square root of each AVE observation variable, which was greater than the correlation coefficients between each AVE observation variable and other observation variables, indicating that each observation variable had a high degree of discrimination [1]. As shown in Table S1 and Table S2, the design of the measurement models was both reliable and valid, which could be used for further structural model fitting analysis.

**Table S1** The factor load, Cronbach’s alpha, average variance extraction (AVE), and composite reliability (CR) values of each variable.

| **Variables** | **Factor load** | **Cronbach’s alpha** | **AVE** | **CR** |
| --- | --- | --- | --- | --- |
| **CRHL** |  | 0.788 | 0.608 | 0.820 |
| Basic knowledge of COVID-19 | 0.613 |  |  |  |
| Prevention behavior knowledge of COVID-19 | 0.838 |  |  |  |
| Protection skills of COVID-19 | 0.864 |  |  |  |
| **GHL** |  | 0.897 | 0.686 | 0.916 |
| Scientific health view | 0.800 |  |  |  |
| Infectious disease prevention and control literacy | 0.732 |  |  |  |
| Chronic disease prevention and control literacy | 0.883 |  |  |  |
| Basic medical literacy | 0.854 |  |  |  |
| Health information literacy | 0.863 |  |  |  |
| **CRIRA** |  | 0.822 | 0.541 | 0.825 |
| Information source authority | 0.697 |  |  |  |
| Information reporting adequacy | 0.768 |  |  |  |
| Information expression intelligibility | 0.752 |  |  |  |
| Information content practicability | 0.722 |  |  |  |

**Table S2** Discriminant validity matrix ^1^.

| **Variables** | **CRHL** | **GHL** | **CRIRA** |
| --- | --- | --- | --- |
| CRHL | **0.780** |  |  |
| GHL | 0.707 | **0.828** |  |
| CRIRA | 0.247 | 0.248 | **0.735** |
| AVE ^2^ | 0.608 | 0.686 | 0.541 |

^1^ The diagonal of the matrix is the square root of AVE of the corresponding variable.

^2^ AVE: average variance extraction.

**Reference**

[1] Hair JF, Black WC, Babin BJ, Anderson RE. Multivariate Data Analysis. 7th ed. Upper Saddle River: Prentice Hall; 2009.
